# Cytokine profile and lymphocyte subsets in type 2 diabetes

**DOI:** 10.1590/1414-431X20155062

**Published:** 2016-03-18

**Authors:** C.O. Francisco, A.M. Catai, S.C.G. Moura-Tonello, L.C.M. Arruda, S.L.B. Lopes, B.G. Benze, A.M. Del Vale, K.C.R. Malmegrim, A.M.O. Leal

**Affiliations:** 1 Departamento de Fisioterapia, Universidade Federal de São Carlos, São Carlos, SP, Brasil; 2 Centro de Terapia Celular, Fundação de Amparo è Pesquisa do Estado de São Paulo, Ribeirão Preto, SP, Brasil; 3 Departamento de Bioquímica e Imunologia, Faculdade de Medicina de Ribeirão Preto, Universidade de São Paulo, Ribeirão Preto, SP, Brasil; 4 Departamento de Medicina, Universidade Federal de São Carlos, São Carlos, SP, Brasil; 5 Departamento de Estatística, Universidade Federal de São Carlos, São Carlos, SP, Brasil; 6 Departamento de Análises Clínicas, Toxicológicas e Bromatológicas, Faculdade de Ciências Farmacêuticas de Ribeirão Preto, Universidade de São Paulo, Ribeirão Preto, SP, Brasil

**Keywords:** Type 2 diabetes mellitus, Cytokines, Lymphocytes, Inflammation

## Abstract

Type 2 diabetes mellitus (T2D) is a metabolic disease with inflammation as an important pathogenic background. However, the pattern of immune cell subsets and the cytokine profile associated with development of T2D are unclear. The objective of this study was to evaluate different components of the immune system in T2D patients' peripheral blood by quantifying the frequency of lymphocyte subsets and intracellular pro- and anti-inflammatory cytokine production by T cells. Clinical data and blood samples were collected from 22 men (51.6±6.3 years old) with T2D and 20 nonsmoking men (49.4±7.6 years old) who were matched for age and sex as control subjects. Glycated hemoglobin, high-sensitivity C-reactive protein concentrations, and the lipid profile were measured by a commercially available automated system. Frequencies of lymphocyte subsets in peripheral blood and intracellular production of interleukin (IL)-4, IL-10, IL-17, tumor necrosis factor-α, and interferon-γ cytokines by CD3^+^ T cells were assessed by flow cytometry. No differences were observed in the frequency of CD19^+^ B cells, CD3^+^CD8^+^ and CD3^+^CD4^+^ T cells, CD16^+^56^+^ NK cells, and CD4^+^CD25^+^Foxp3^+^ T regulatory cells in patients with T2D compared with controls. The numbers of IL-10- and IL-17-producing CD3^+^ T cells were significantly higher in patients with T2D than in controls (P<0.05). The frequency of interferon-γ-producing CD3^+^ T cells was positively correlated with body mass index (r=0.59; P=0.01). In conclusion, this study shows increased numbers of circulating IL-10- and IL-17-producing CD3^+^ T cells in patients with T2D, suggesting that these cytokines are involved in the immune pathology of this disease.

## Introduction

Type 2 diabetes mellitus (T2D) is currently recognized as a chronic inflammatory disease with involvement of pro-inflammatory cytokines and immune cells, including B and T-cell subsets as pathogenic mediators (1 2 3). There is evidence from animal models and human studies that functional changes and alterations in the cytokine profiles produced by these cells, especially by the specific pro-inflammatory T helper 1 (Th1) subset of CD4+ T cells, are associated with the pathogenesis of T2D (2,4). Obesity is usually linked to the inflammatory process observed in T2D. Increased adipose tissue is an important scenario and source of inflammatory cells and mediators. This leads to insulin resistance and ultimately to hyperglycemia by exhaustion of the adaptive capacity of pancreatic beta cells. In addition, an abundance of nutrients may also directly induce local inflammation in the pancreatic islets (3). However, the pattern of immune cell subsets and the cytokine profile associated with development of T2D remain controversial. This is partly because of the complexity of the immune system network and partly owing to the different methods used to address the problem. Nevertheless, this is an important issue because investigation of the relationship between T2D and the immune system may lead to development of new and more efficient treatments for this disease.

The objective of this study was to evaluate different components of the immune system in peripheral blood of patients with T2D by quantifying the frequency of lymphocyte subsets and intracellular production of pro- and anti-inflammatory cytokines by T cells.

## Material and Methods

This case-controlled study was approved by the Ethics Committee of the Universidade Federal de São Carlos (#412/2010). Twenty-two men with T2D (51.6±6.3 years old) and 20 men matched for age (49.4±7.6 years old) and sex (control subjects) participated in the study after providing written informed consent. All individuals were nonsmokers.

Type 2 diabetes mellitus was diagnosed according to the recommendations of the American Diabetes Association (5). Body mass index (BMI) was not matched between diabetes mellitus (DM) and control subjects because obesity is an important hallmark of T2D, even though it is associated with inflammation *per se*.

Individuals with DM were medicated with metformin, gliclazide, and/or insulin for hyperglycemia, angiotensin-converting enzyme inhibitors for hypertension, and simvastatin for dyslipidemia. Exclusion criteria were smoking, anemia, alcoholism, use of anti-inflammatory drugs or inflammatory and respiratory diseases, congestive heart failure, and ischemic coronary disease (6).

On the experimental day, after 10-12 h of fasting, the subjects underwent anthropometrical evaluation and blood samples were drawn. Glycated hemoglobin (HbA1c), high-sensitivity C-reactive protein (CRP) concentrations, and the lipid profile were measured using an Advia 1800 Chemistry System (Siemens, USA).

Peripheral blood mononuclear cells (PBMCs) were isolated by Ficoll-Hypaque gradient centrifugation (GE Healthcare, Sweden). After separation and washing, PBMCs were suspended in fetal bovine serum with 10% of dimethyl sulfoxide (Sigma-Aldrich, Germany) and frozen in liquid nitrogen.

### Flow cytometry

Cells (1×10^6^ PBMCs) were incubated for 10 min at room temperature with 5 μL of the following fluorochrome-conjugated antibodies: CD3-FITC, CD19-PE, CD8-PE, CD4-APC, CD16,56-PE, CD4-FITC and/or CD25-APC (all from BD Biosciences, USA). The cells were washed with FACS buffer (phosphate-buffered saline, 0.2% fetal bovine serum, 0.02% sodium azide) and centrifuged for 5 min at 500 ×*g*. For analysis of FoxP3 expression, the cells were permeabilized by 300 μL of FACS permeabilizing solution (BD Biosciences) for 10 min at room temperature in the dark, washed, and labeled with 5 μL FoxP3-PE antibody for 10 min at room temperature (eBioscience, USA). Isotype controls were used for each staining procedure as negative controls and for compensation of fluorescence. Thirty thousand events were acquired for each cell subset analysis and 100,000 events for regulatory T-cell analysis, using a FACSort flow cytometer (BD Biosciences). The CD127lo marker was not evaluated in this study.

### Measurement of cytokine production

To measure intracellular cytokines, 1×10^7^ PBMCs were re-suspended in tissue culture medium (RPMI, Sigma-Aldrich) and incubated with 25 µL/mL 12-myristrate 13-acetate (Sigma-Aldrich) and 1 µg/ml ionomycin (Sigma-Aldrich) in the presence of brefeldin for 6 h. After harvest, cells were stained with anti-CD3-FITC for 20 min in the dark. Cells were then treated with FACS lysing solution and with FACS permeabilization solution (both from BD Biosciences). Lastly, cells were incubated with anti-interleukin (IL)-4, anti-IL-10, anti-IL17, anti-tumor necrosis factor (TNF)-α, and anti-interferon (IFN)-γ fluorochrome-conjugated antibodies for 30 min in the dark and re-suspended in PBS with 1% of paraformaldehyde (Sigma-Aldrich). All analyses were performed by a FACSort flow cytometer (BD Bioscience) and 100,000 cells were acquired for each sample. Isotype controls were used for each staining procedure as negative controls and for compensation of fluorescence.

### Statistical analysis

Results are reported as means±SD or median and range for parametric and nonparametric tests, respectively. The Shapiro-Wilk test was used to test normality of the distribution of data. Group differences in variables were compared using Student's *t*-test or the Mann-Whitney test. Relationships between variables were determined using the Pearson correlation test. P<0.05 was considered to be statistically significant. All analyses were performed using the SPSS software program version 17.0 for Windows (IBM, USA).

## Results

The lipid profile (triglycerides, high density lipoprotein, low density lipoprotein, and total cholesterol) and plasma CRP levels were similar in individuals with T2D and controls. As expected, BMI and HbA1c levels were significantly higher in individuals with T2D than in controls (Table 1).

**Table:**
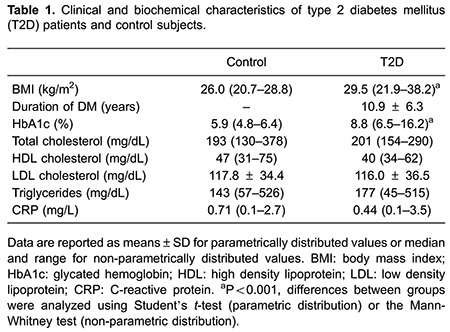

**Figure 1:**
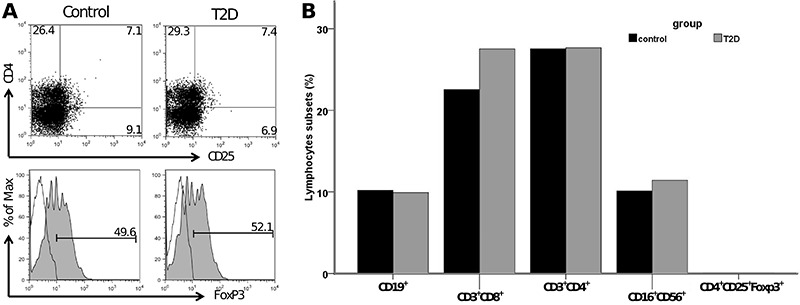
Frequency of lymphocyte subsets in the control group (control) and type 2 diabetes group (T2D). *A*, Gating strategy and staining of CD4^+^CD25^+^ (upper panel) and intracellular expression of FoxP3 (lower panel) in lymphocytes from one representative patient. *B*, Frequencies of peripheral CD19^+^ B cells, CD3^+^CD8^+^ and CD3^+^CD4^+^ T cells, CD16^+^56^+^ NK cells, and CD4^+^CD25^+^Foxp3^+^ T regulatory cells.

No differences were observed in the frequency of CD19^+^ B cells, CD3^+^CD8^+^ and CD3^+^CD4^+^ T cells, CD16^+^56^+^ NK cells, and CD4^+^CD25^+^Foxp3^+^ T regulatory cells in peripheral blood of T2D patients compared with controls (Figure 1).

**Figure 2:**
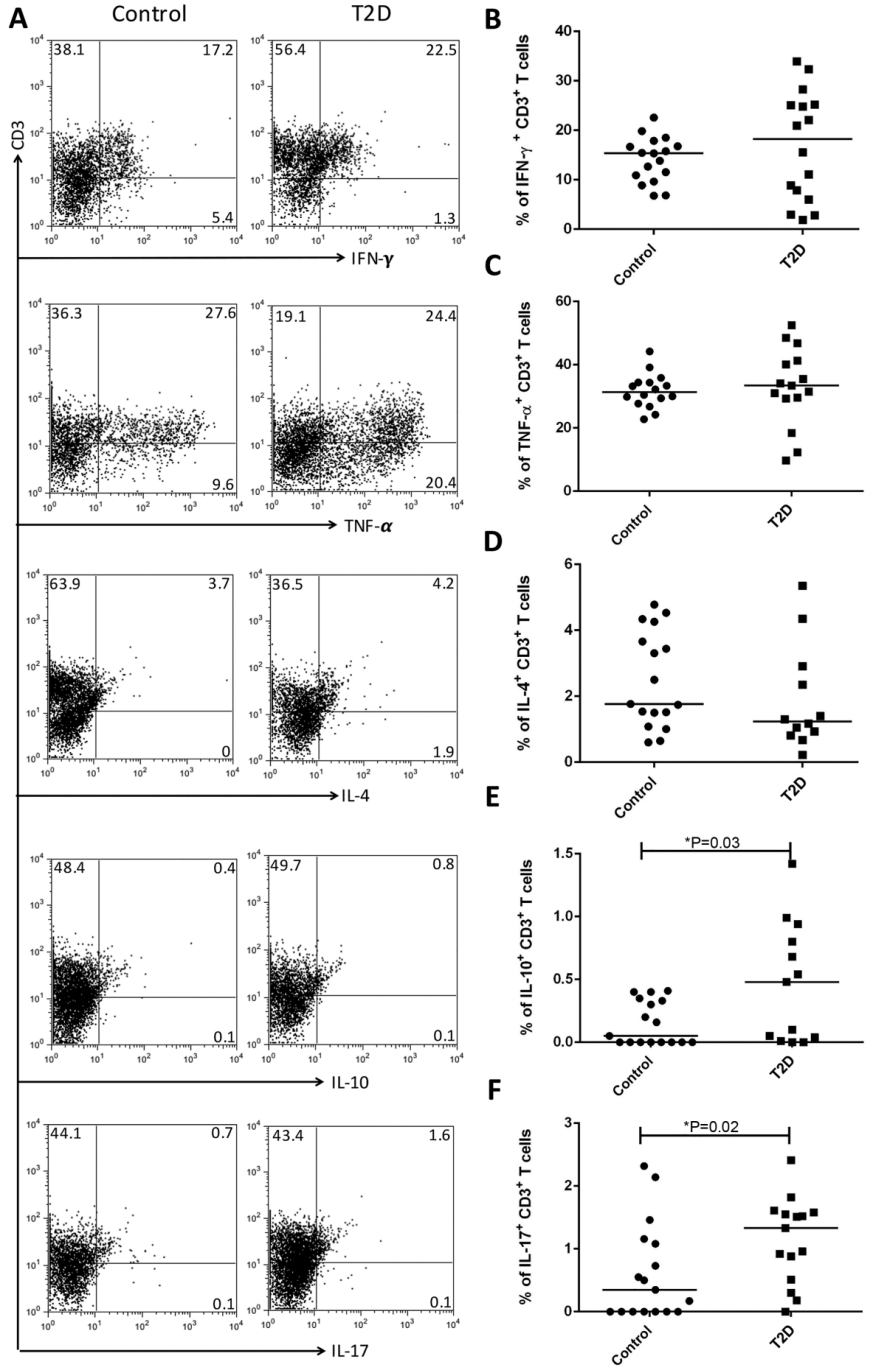
Frequency of cytokine-producing T cells in the control group and type 2 diabetes group (T2D). *A*, Gating strategy and expression of anti-interferon (IFN)-γ, anti-tumor necrosis factor (TNF)-α, interleukin (IL)-4, IL-10, and IL-17 by CD3^+^ T cells from one representative patient. Frequencies of peripheral (*B*) IFN-γ, (*C*) TNF-α-, (*D*) IL-4-, (*E*) IL-10-, and (*F*) IL-17-producing CD3^+^ T cells. *P<0.05 [Student's *t*-test (parametric distribution) or the Mann-Whitney test (non-parametric distribution)].

The frequencies of peripheral blood lymphocyte subsets and intracellular cytokine production of IL-4, IL-10, IL-17, TNF-α, and IFN-γ by CD3^+^ T cells are shown in Figure 2. The frequencies of IL-10- and IL-17-producing CD3^+^ T cells were significantly higher in patients with T2D than in controls (P<0.05). There were no differences in the percentage of other cytokine-producing T cells. The frequency of IFN-γ-producing CD3^+^ T cells was positively correlated with BMI (r=0.59; P=0.01). No other correlations were observed between metabolic variables or the duration of T2D and immunological evaluations.

## Discussion

In this study, we showed increased frequencies of IL-10- and IL-17-producing CD3^+^ T cells in T2D patients. However, no difference in the frequency of lymphocyte subsets was observed. Increased levels of IL-17 are associated with inflammation and insulin resistance in T2D patients (7 8 9).

A parallel increase in the production of these two cytokines with apparent opposing functions could indicate an attempt, although unsuccessful, to maintain metabolic and immune homeostasis. IL-10 has been recognized as a Th2 cytokine with anti-inflammatory and immunoregulatory properties. However, IL-10 can also have pro-inflammatory effects and complex interactions with diverse immune cell subsets, resulting in modulation of adaptive immunity (10). Furthermore, we cannot discount the possible presence of CD3+ T cells co-expressing IL-17 and IL-10/IFN-γ because of technical limitations. These subtypes of Th17 cells can achieve IL-10 expression and become immunosuppressive (10), as well as achieve IFN-γ expression and become more inflammatory (11,12); however, the role of these subpopulations in T2D remains unclear.

The present data do not support previous studies that demonstrated increased numbers of Th1 cytokine-producing cells in peripheral blood of T2D (2,4). However, in our study, there was a positive correlation between the frequency of IFN-γ-producing CD3^+^ T cells and BMI, as previously described (13).

We found no differences in the frequency of lymphocyte subsets in T2D patients compared with controls. Notably, previous studies have reported a decrease in the frequency of percentage of CD4^+^CD25^hi^ T cells and an increase in Th22 cells in patients with T2D (9,14).

The pattern of immune cell subsets and the cytokine profile associated with development of T2D remain controversial. Unfortunately, demonstration of immune alterations in animal models of T2D may not mirror the findings in diabetic patients (2). In addition to species-specific immune responses and the methodological differences among studies, the complexity and dynamicity that characterize the immune response may contribute to the discrepant findings among studies. Moreover, notably, peripheral blood may present with different patterns of immune responses than local inflammation found in adipose tissue and in other insulin-targeted tissues.

Our study included patients with diseases associated with T2D (obesity, hypertension, and dyslipidemia) and these volunteers were on drug treatment. These diseases could be associated with inflammation *per se*. The design of the present study did not allow evaluation of how these factors affected our results. Our results represented the inflammatory process in patients with T2D.

With regard to medicine, metformin may have anti-inflammatory actions in addition to its metabolic effects, which could interfere in inflammatory signaling factors (15). The effects of this drug*per se* on the immune response of patients with T2D cannot be ruled out. A recent study compared the cytokine profile in newly-diagnosed T2D patients at pre- and post-metformin treatment and showed decreased serum IL-17 levels, but no changes in IFN-γ levels (8).

The duration of DM could influence the inflammatory process. Nevertheless, the cross-sectional design adopted in our study did not allow evaluation of this factor. In addition, defining the duration of T2D is highly imprecise because few individuals are aware of this condition.

In conclusion, this study showed increased numbers of circulating IL-10- and IL-17-producing CD3^+^ T cells in T2D patients. Our findings indicate that these cytokines may be involved in the immune pathology of T2D.
